# Respiratory Follow Up of the Premature Neonates—Rationale and Practical Issues

**DOI:** 10.3390/jcm11061746

**Published:** 2022-03-21

**Authors:** Raluca Daniela Bogdan, Roxana Elena Bohiltea, Adrian Ioan Toma

**Affiliations:** 1 Pediatrics Department, Medicover Hospital, Str. Pechea No. 8, Sector 1, 031056 Bucharest, Romania; ralbgd@gmail.com; 2 Department of Obstetrics and Gynecology, Carol Davila University of Medicine and Pharmacy, Bd Eroii Sanitari Nr 8, 050471 Bucharest, Romania; 3 Neonatology Department, Life Memorial Hospital, Calea Grivitei No. 365, Sector 1, 010719 Bucharest, Romania; 4 Faculty of Medicine, University “Titu Maiorescu”, Str. Gh Petrascu 67, Sector 3, 031593 Bucharest, Romania

**Keywords:** premature neonates, respiratory follow-up, respiratory tract infections, wheezing, asthma, pulmonary function tests, late preterm neonates

## Abstract

The aim of the review was to present the state of knowledge about the respiratory pathology in former premature neonates (children that were born preterm—before 37 weeks of gestation—and are examined and evaluated after 40 weeks corrected age) other than chronic lung disease, in order to provide reasons for a respiratory follow-up program for this category of patients. After a search of the current evidence, we found that premature infants are prone to long-term respiratory consequences due to several reasons: development of the lung outside of the uterus, leading to dysmaturation of the structures, pulmonary pathology due to immaturity, infectious agents or mechanical ventilation and deficient control of breathing. The medium- to long-term respiratory consequences of being born before term are represented by an increased risk of respiratory infections (especially viral) during the first years of life, a risk of recurrent wheezing and asthma and a decrease in pulmonary volumes and airway flows. Late preterm infants have risks of pulmonary long-term consequences similar to other former premature infants. Due to all the above risks, premature neonates should be followed in an organized fashion, being examined at regular time intervals from discharge from the maternity hospital until adulthood—this could lead to an early detection of the risks and preventive therapies in order to improve their prognosis and assure a normal and productive life. The difficulties related to establishing such programs are represented by the insufficient standardization of the data gathering forms, clinical examinations and lung function tests, but it is our belief that if more premature infants are followed, the experience will allow standards to be established in these fields and the methods of data gathering and evaluation to be unified.

## 1. Introduction

Due to improvements in prenatal care [1] and neonatal intensive care, there has been an increased survival of premature neonates of low gestational ages during the last decades [2]. There has also been observed a phenomenon of an increase in the number of late preterm neonates due to an increase in the number of iterative caesarean sections, an increase in the number of children born after human-assisted reproduction techniques and an increase in the age of the mothers [3]. 

Considering this, there has been an increased interest in the follow-up of these categories of neonates, with the following objectives [4]:

- Support for the child—early identification of concerns and early intervention.
- Support for the families—monitoring normal/abnormal growth and development and helping in the decisions about the future of the child.
- Support for the healthcare system—resource allocation and planning of health care services.

The follow-up programs have focused mainly of the neurological outcomes of the NICU graduates, especially premature infants [4,5], although, the respiratory follow-up component is mentioned in guidelines for establishing a follow-up program for high-risk neonates [4] There could be several reasons as to why the respiratory follow-up programs are not as widespread as the neurologic ones. First, even if premature infants are prone to respiratory problems due to the immaturity of their respiratory systems [6] and the respiratory pathology represents the main cause of admission in the neonatal intensive care unit [6,7], the research in the field of the respiratory consequences of prematurity is not so abundant and well known [8,9] as the research in the field of neurological consequences and neurological follow-up [4,5,10,11,12]. The main aim of this paper is to raise the awareness about this pathology by presenting, in a structured manner, the present state of knowledge about the respiratory outcomes of former premature infants. Second, the frame for the respiratory follow-up program—forms for data gathering, clinical examinations and functional tests—is not so well established and standardized as in the case of the respiratory follow-up [4,5,9,13,14,15,16,17]. These issues will be discussed in detail in the final chapter of this paper.

## 2. Reasons for Establishing a Respiratory Follow-Up Program

A respiratory follow-up program for NICU graduates seems more than appropriate, for several reasons (Table 1).

**Figure 1:**
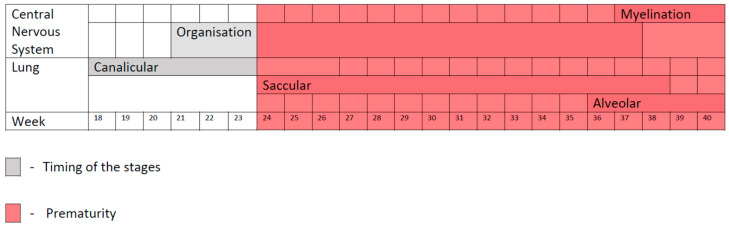
Central nervous system and lung development.

First, as in the case of the development of the central nervous system [10], the development of the lung takes place during the whole of the intrauterine life and continues for a long time after the delivery [18,19]. As you can see in Figure 1, at the moment of a premature delivery, both the CNS and lungs are immature. From the CNS point of view, premature neonates are born in the middle of the organization phase and at the beginning of the synaptogenesis—the extrauterine occurrence of a process of development that should have occurred in utero will lead to phenomena of dysmaturation at the level of the CNS [11,12]. This is also the case regarding the development of the lung: premature neonates are born at the end of the canalicular stage (16–26 weeks) and during the saccular stage (24–38 weeks) and at the beginning of the alveolar phase [18]. As a reminder from the medical literature, the process of formation of the lung has four components—development of the conducting airways, development of the gas-exchange surface area (epithelium), the development of an effective vascular system and the production of surfactant—that will assure the stability of the alveoli at the end of the expiratory phase [18]. During the canalicular stage, the formation of the airways is almost finished and the formation of the respiratory epithelium begins, with the differentiation of the type I and II pneumocytes and the beginning of the formation of the air–blood barrier (though still an immature one); also, the secretion of surfactant begins [18]. During the saccular stage, an intermediate stage [18], there is an expansion of the gas-exchange area by formation of the saccule and primary alveolar septa, with a double layer capillary bed—an immature gas-exchange surface [18]. At the time of birth, the alveoli begin to form [18,19]. Thus, during the extrauterine life of a premature neonate, all the four components of the lung: airways, surface area, vascular component and surfactant system are in full development and all could be affected, resulting in dysfunction of the airways, alveolar formation and vascular components, as will be further detailed.

The second issue regarding the long-term risks for the respiratory system of a premature infant is represented by the specific pulmonary pathology of the neonate related either to immaturity, specific to the premature neonate: respiratory distress syndrome [20], transient tachypnea of the newborn [21]; or pulmonary pathology non-specific to the premature infant (congenital pneumonia [22] or air leak syndromes [23]), the pulmonary diseases being the most frequent diagnosis of admission in the neonatal intensive care unit. All these diseases affect an immature lung and, thus, short- and long-term consequences will appear [6,24,25,26,27].

A third reason for the long-term risks related to the respiratory system are the issues related to control of breathing (apnea, both obstructive and central) [8]. This topic will not be covered in the present review. The control of breathing is a neurologic process, processes occurring in the brainstem respiratory center and feedback loops from the breathing control receptors [28]. Though these processes could have an obstructive component, the vast majority of control of breathing problems in premature infants and former premature babies are related to CNS pathology [8,28]. In addition, premature infants with control of breathing problems (apnea, hypoventilation) are already included in the neurologic follow-up program due to their neurologic pathology [4,5]. This is why we decided not to address this type of problem in our review.

- Based on the current knowledge, the respiratory consequences of prematurity can be classified in:
- Chronic lung disease of the premature infants (bronchopulmonary dysplasia);
- Risks of respiratory tract infections;
- Risk of wheezing and asthma;
- Modification of the pulmonary function tests.

The last three topics—except BPD—will be covered in the present review. Bronchopulmonary dysplasia represents, indeed, the main pulmonary pathology of the extremely premature neonates [29,30]. There is already a vast medical literature on this subject [31,32,33,34], and it is already established that a baby with this diagnosis will benefit from respiratory follow-up [4]. Our aim was to point out that there are other categories of premature infants at risk for medium- and long-term respiratory pathology that would benefit from being included in a respiratory follow-up program. A special section will be dedicated to late preterm neonates. In the end, the criteria for inclusion in a follow-up program and a respiratory follow-up schedule will be proposed.

**Figure 2:**
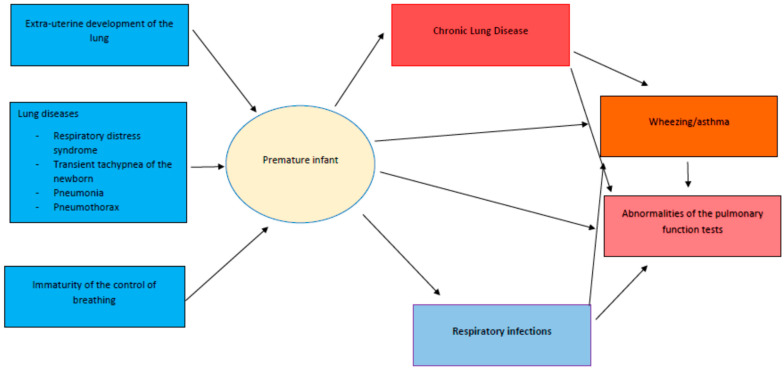
Respiratory consequences of prematurity.

Figure 2 presents the respiratory risks for premature infants and the respiratory consequences of prematurity.

## 3. Risks of Respiratory Infections in the Former Premature Newborns

Former premature neonates are at risk for respiratory tract infections during the first years of life. In a study published in 2003, Doyle et al. found that ELBW (extremely low birthweight neonates—neonates with birthweight < 1000 g [35]) have significantly more hospital readmissions than children with birthweights more than 2499 g [36]. The findings showed that, during three time intervals: 1980–1982, 1991–1992 and 1997, the readmission rate was significantly higher and constant for ELBW neonates, around 50% (52–66%) [36]. According to the same group, respiratory illnesses were the most common cause of readmission in this group of patients (55–69%) [36]. This fact is confirmed also by other studies, which found that 2 out of 3 of the readmissions of premature infants were of respiratory cause [37]. The number of days of hospitalization was higher in the ELBW infants compared with their NBW controls [36] (more than 2 days versus around 1.3 days). The tendency for higher rates of hospitalization is maintained in this category of neonates until 8–10 years of age [38].

The duration of mechanical ventilation and the birthweight seem to be predictive of the occurrence of respiratory infections in former premature neonates. In a study published in 2018, MacBean and co-workers identified three clusters related to the risk of low respiratory tract infections—48% of the neonates ventilated less than 5 days had a lower respiratory tract infection during the first year, compared with 65% of those ventilated more than 5 days with birthweights more than 882 g and 100% of those ventilated more than 5 days with a birthweight less than 882 g [39]. The pathogens also differed—the second cluster had more cases of respiratory syncytial virus infections and cluster three had more cases of rhinovirus infections [39].

As mentioned above, two pathogens account for most of the respiratory infections in the former premature neonates: respiratory syncytial virus (RSV) and human rhinovirus (HRV). Even if RSV has been studied in more detail, rhinoviruses make up an important part of the pathology of preterm neonates. In a cohort study performed in Argentina, 65% of the VLBW (very low birthweight neonates—neonates with birthweight < 1500 g [35]) infants had respiratory tract infections caused by human rhinoviruses [40]. In the same study, 40% of the infants with bronchiolitis had an HRV infection and only 7% RSV [40] (it is worth mentioning that the study population did not receive palivizumab prophylaxis). The risk was higher for infants with bronchopulmonary dysplasia [40]. Immunology studies [41,42] have shown the effects of the HRV at different levels in the lung. Thus, after HRV infection in young children, there is an enhanced production of remodeling by Th2 (T helper 2) (IL (interleukin) 4 and IL 13) and Th17 (T helper 17) cells (IL 17 [42], growth factors for the airways (HGF (Hepatic Growth Factor) and TGFα (transforming Growth Factor α)) and modified ratios in other factors (lower MMP-9 (matrix metalloproteinase 9/TIMP-2 (tissue inhibitor metalloproteinase)) and MMP-2 (matrix metalloproteinase 2)/TIMP-2 ratios and increased MMP-10 (matrix metalloproteinase 10)/TIMP-1 (tissue inhibitor metalloproteinase 1 ratios)) [41]. Premature infants had, according to the same study, a decreased baseline MMP-9/TMP-2 ratio [41]. The implications of these findings are not fully understood yet, but remodeling factors in the airway are implicated in apoptosis, healing, fibrosis and other processes, and abnormalities in these factors could play a role in the occurrence of airway disorders in this group of children. More than this, another study found that during HRV infections, severely premature children (gestational age < 32 weeks, according to the authors) have an increased secretion of the cytokines produced [42]. According to the reference quoted [42], the premature infants with an increased airway secretion of Th2 and Th17 cytokines during RV infections experienced more respiratory morbidity during the first two years of life. Premature children with high nasal IL4 levels during natural RV infections were associated with 8 times increased odds of having at least one PICU admission during the first two years of life. High RV-induced IL17 was also associated with 5 times increased odds of a history of PICU admission during early childhood [42].

Prematurity represents a risk factor for both the infection with RSV and the most severe form of it—bronchiolitis. Thus, a premature infant has a seven-fold risk factor for acute viral bronchiolitis during the first RSV season after their birth [43]. There are many risk factors for the appearance of severe RSV infections in this high-risk group and they have been investigated in studies [43,44]. In a study published by our group, we identified as independent risk factors for the appearance of RSV infections, the familial atopy and lower gestational age [45]. The long term consequences of acute RSV bronchiolitis in the premature neonate are represented by an increase in the risk of wheezing [46] and possibly asthma in the future [47]. Considering this, there is a debate if palivizumab prophylaxis improves the outcome of the premature infant regarding the risk of recurrent wheezing or asthma. The issue is not clear yet. A study published in 2013, stated that there was a reduction of 65% in the total number of wheezing days during the first year of life in the RSV prevention group, and the proportion of patients with wheezing was 10% lower in the palivizumab group than the placebo group [46]. The patients were premature infants, 33–35 weeks gestational age, who received prophylaxis during the first year of life. This study found also that a small proportion of the children with acute respiratory infections had RSV, and the coinfection rate was higher in the palivizumab prophylaxis group, probably due to the absence of local lung environment modifications secondary to RSV infections that precluded other pathogens from infecting the lung [46]. The same cohort was reassessed for the risk of asthma at the age of 6 years [48]. There was observed a decreased number of cases of asthma self-reported by the parents in the prevention group compared to the placebo group [48], but no difference in the cases diagnosed by the physicians at the age of 6. There were no differences in the pulmonary function tests [48]. Another interesting finding was that while at the end of the first year the proportion of children with wheezing was higher in the placebo group than in the prevention group, the proportion of new cases of wheezing after the first year was the same in the two groups [48]. Another study published by a Japanese group found that there was a reduction in the recurrent wheezing diagnosed at 6 years by the physician; the study has, though, several limitations because of the confounders present at the beginning of the research in the two groups (there were statistically significant differences between the two groups regarding the gestational age, smokers at home and family history of allergy) [49]. It is interesting to notice that the RSV prophylaxis decreases the risk of asthma in non-atopic children but not in atopic children [50]; probably, atopy and RSV infections act as independent risk factors and atopy per se is sufficient to increase the risk of asthma in the former preterm infants. It seems that the mechanism by which RSV acts in increasing the risk of recurrent wheezing and asthma is represented by an induction of a predominantly Th2 response to respiratory allergens [49]. Finally, there are questions about the cost-efficiency of RSV prophylaxis in premature neonates [51], and even its long-term efficiency. The reasons for this are multiple [52]. Thus, there have not been observed long-term effects on respiratory morbidity, airway reactivity or allergic parameters [52]. Additionally, the levels of the cytokines of the cells involved in the abnormal immune response in asthma Th2 and Th17 were higher [52].

**Figure 3:**
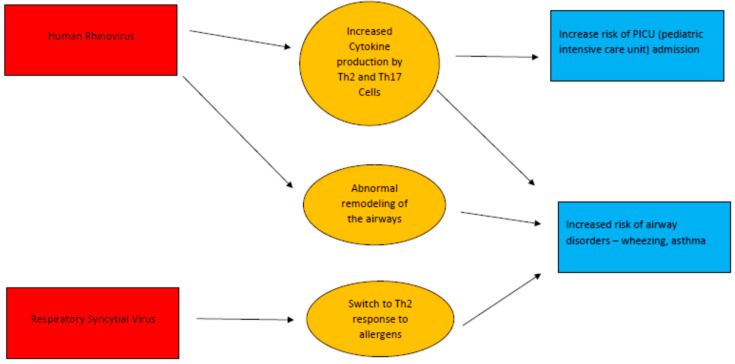
Long term pulmonary consequences of the respiratory tract infections in the premature infants [40,41,42,49,50].

Figure 3 summarizes the mechanisms and the risks for long-term pulmonary consequences of the respiratory tract infections in the former premature neonates.

## 4. Relationship between Premature Birth and Wheezing Disorders

A large amount of the medical literature addresses the topic of bronchial hyperresponsiveness and wheezing disorders directly linked to premature birth. Furthermore a number of reviews have given more statistical power to this association.

A systematic review published in January 2014 [53] found a 1.71 increased risk of wheezing disorders in preterm babies with an inverse correlation between gestational age at birth and the risk of wheezing. The authors acknowledge the fact that there was a considerable heterogenicity among studies, probably due to the fact that variability exists among doctors and parents in recognizing and defining wheezing disorders, and also because several determinants of premature delivery such as pollution, maternal smoking and hygiene are factors that have been previously linked independently to wheezing disorders.

Such findings support the idea that the relationship between asthma-like conditions and prematurity is a complex, multifactorial one and one should be aware of as many of these aspects as possible in order to be able to find the best medical approach in a former premature child with recurrent wheezing.

Another review, published in 2018 [54], has a more comprehensive approach, addressing the physio-pathologic mechanisms that underlie the relationship between prematurity and bronchial hyperresponsiveness. Thus two scenarios are formulated regarding physio-pathologic mechanisms (Table 2). The classic “structural disease scenario” considered a direct consequence of injuries related to Infant Respiratory Distress Syndrome and bronchopulmonary dysplasia in which altered elastic and fibrous networks caused by hyperoxia and positive pressure ventilation leads to a loss of elastic recoil and a lack of antagonism to bronchoconstriction. The clinical picture caused by this mechanism differs from that of classic asthma in regard to the lack of an eosinophilic response and less response to bronchodilator medication. The second “active disease scenario” is still controversial, requiring more data to link bronchial hyperresponsiveness to markers of airway inflammation. The classic eosinophilic pattern of bronchial asthma is missing. Recently, a neutrophilic-type inflammation has been described as has a connection between prematurity and the development of Chronic Obstructive Pulmonary Disease later in life [55].

However there is an increasing amount of evidence regarding prematurity and prematurity-related exposers and the risk of developing atopy and other inflammatory disorders.

In a perspective-type article, a group of immunologists drew attention to several factors altering the inflammatory responses in premature babies [56].

In consequence, the maturational process of the immune system of the fetus—a highly complex process—is altered in the case of a premature birth. Soon after birth, the premature neonate establishes a large number of class-switched B cells, but their IG G and IG A production maintains fetus-like characteristics such as small amounts of antibody production and low affinity to antigens, as seen in response to vaccination. Furthermore, there is a lack of maternally-transmitted passive immunity through antibodies crossing the placenta in the third trimester with an amount of maternal antibodies in VLBW babies of just 10–20% of the amount found in a term neonate. As well as reducing the immune protection of the baby, there seems to be a more intricate relationship with idiotypic control of B and T cell receptors, thus contributing to the adaptative immune responses [57,58]. Age-dependent failure to mount an effective Th1 response and a shift towards Th2 type was incriminated for atopy risk in children exposed to certain pathogens [59].

A number of extrinsic factors have been associated with prematurity and/or asthma development risk.

Stress has long been suspected to be both a cause for premature labor and a risk factor for developing abnormal immunologic responses in the offspring, thus leading to atopy and asthma. A large cohort study of a potent stressful factor, an electricity system failure for 45 days during an ice storm in Quebec in 1998, found larger proportions of premature birth and immunological dysregulation in the offspring of women pregnant at that time [60].

Another mechanism for developing immunologic dysregulation might be premature, stimulation of the chemosensory system in a baby born prematurely. This hypothesis is sustained by the scientifically proven fact that chemosensory system stimulation is an important mechanism of bacterial recognition [61].

A number of clinical trials yielded interesting results, finding statistically significant associations between atopy and/or wheezing on the one hand and, on the other hand, prematurity-associated conditions such as maternal diabetes, mechanical ventilation and antibiotic use rather than with prematurity per se [62,63,64].

A number of indices related to respiratory distress in the neonatal period have been associated with asthma in medical trials. Thus, increased oxygen exposure in the first days of life, a higher number of hypoxic episodes and a lower oxygen saturation level have been linked to a higher risk of asthma in a cohort study of premature babies < 28 weeks gestation [65]. Other studies postulated that oxidative stress caused by hyperoxia following hypoxic episodes or independently might cause oxidative stress and lung injury in premature babies [66,67].

Overall, we can conclude there is a definite association between prematurity and asthma-like features. Although some additional factors (e.g., infections, mechanical ventilation, oxygen exposure, family history) seem to influence this relationship; when evaluating a premature baby, one must also consider not just their gestational age or birthweight but also all the additional perinatal factors that will definitely influence their future morbidity in the short as well as the long term.

## 5. Lung Function Follow-Up in the Former Premature Neonates

Evaluating lung function is an interesting topic for researchers as well as for clinicians dealing with former premature babies presenting with respiratory morbidity later in life. However, measuring lung function can be challenging, especially due to the lack of proper standardized techniques and the lack of cooperation from children, especially those who are very young or with neuropsychiatric disorders. Efforts are being conducted by a united task force of both the American Respiratory Society (ARS) and European Respiratory Society (ERS) toward proper standardized techniques of evaluating pulmonary function in children.

For children older than 5 years, pulmonary function can be investigated using spirometry—a procedure last updated in a paper published in 2019 in regard to proper investigation technique, investigation conditions and events that may alter a respiratory curve, thus diminishing measurement accuracy [68].

For the pulmonary function of infants, the matter is more complicated. Recommendations regarding standardizing tidal breath analysis were issued by the ARS/ERS Task Force in 2000. The need for eliminating all sources of leak, minimizing dead space equipment and using a device with proper linearity and frequency response was emphasized. The authors admit there is still a challenge to proper identify the beginning and end of inspiration and expiration and establishing appropriate reference standards [69].

In 2005, more recommendations were issued regarding the raised volume rapid thoracoabdominal compression (RVRTC) maneuver. In spite of considerable efforts made to develop and standardize this technique, and the fact that it provides capabilities of measuring an extended range of pulmonary volumes, its use has been limited to a few specialized laboratories throughout the world [70].

Whole body plethysmography is another method used in research settings for measuring lung volume, airway resistance and functional residual capacity [71]. Although some efforts have been made by the ATS/ERS Task Force [72], this method lacks appropriate standardization.

Table 3 summarizes the different methods for evaluating pulmonary function in children.

The multiple breath washout test has been rediscovered lately as a valuable tool for evaluating functional residual capacity and peripheric airway disfunction [73]. There are still some technical issues causing inconsistent results among different working groups attributed to the use of different measuring devices [74].

The method has its undisputable use for measuring lung function in children with cystic fibrosis. In measuring lung function for children with chronic lung disease, wheezing and asthma, the method has not yet proven its efficacy or fails to provide consistent result [75].

In spite of all these difficulties, there is a growing amount of data on pulmonary function modifications in a former premature baby.

Thus, in an article published in 2009, follow-up data in a randomized trial population of former premature babies showed an alteration in respiratory function at 11–14 years that manifested through lower volumes on spirometry more frequently in the male population [76]. Another study published in 2017, performed a follow-up on children born before 26 weeks of gestation by measuring lung volumes at 1 year and 11–13 years of age using the helium dilution technique and plethysmography. The study showed lower forced expiratory volumes compared to normal values. Moreover, those who were smokers at age 18 had airway function that further deteriorated in time compared to non-smokers. The obstructive pattern in children also increased over time [77].

Lung function using spirometry was also assessed in a cohort of school-age children who were born at 22–26 weeks gestation. Researchers found lower forced vital capacity (FVC) and forced expiratory volume in one second (FEV1) in former premature babies compared to their normal controls. Asthma manifestations were also found in a greater proportion in the formerly premature baby group [78].

A regional prospective cohort study conducted in Norway compared tidal respiratory function of babies born before 28 weeks gestation with or without a bronchopulmonary dysplasia (BPD) diagnosis to that of term babies using electromagnetic inductance plethysmography. Researchers aimed to study a normal breathing pattern with no interference from using a mask or performing a forced ventilation. Lung volumes of premature born babies measured at term equivalent age were significantly lower when compared to those of a control group of term born infants [79].

A cohort study of 164 former premature babies, children that were born preterm (before 37 weeks of gestation) and are examined or evaluated after 40 weeks corrected age, published in 2021, found a worsening of respiratory function over time as measured by spirometry at 3–6 years of age, 7–11 years and 12–20 years. Mechanical ventilation, postnatal steroids and a maternal history of atopy and asthma were associated with a more adverse outcome [80].

A management technique for improving respiratory function in preterm babies (GA < 32 weeks) was proposed by an article, which has been just approved for publishing, that found that feeding premature babies protein rich formula after discharge will improve lung function and diminish airway inflammation as measured through exhaled NO measured at 6 years of age [81]

In conclusion, prematurity represents a disturbing factor for the developing lung. Tests that are standardized and have been used for a long time such as spirometry and exhaled NO should be considered as soon as the child is old enough to cooperate. The others are worth being considered as possible diagnostic tools as they cross the line from research to clinical practice devices. Future technological development will for sure enable us to have more reliable clinical diagnostic devices.

## 6. A Neglected Category—Late Preterm Newborns

Late preterm infants are defined as infants born between 34 weeks and 36 6/7 weeks gestation [3]. They represent 7–9% of the number of neonates born in the US and 70% of the premature infants [3,82]. The studies showed that this category of neonates is at risk for more complications in the neonatal period: the need for resuscitation, thermal instability, respiratory distress, feeding problems, hyperbilirubinemia and an increased risk of death compared to term neonates [3,82].

These infants are also at risk for medium- and long-term health problems, both neurologic and respiratory [2]. The risk of respiratory consequences is due, according to the present medical literature, to developmental and infectious factors [82]:

- The late preterm neonates are born during the end of the saccular phase and the beginning of the alveolar phase of lung development [82]. Two processes occur simultaneously during this phase: the formation of the alveoli and the tethering of the airways [82,83]. The abnormal formation of the alveoli and the deficient tethering of the airways result in airway dysfunction and the risk of lung infections [82,83]
- There is an immune deficit at the level of the T lymphocytes, a deficient pulmonary cytotoxic lymphocyte response, leading to the attempt to clean the infection by the macrophages and neutrophils [84]. The infection is resolved, but this results in the damage to epithelial cells and abnormal activation of the Th2 lymphocytes, increasing the risk of recurrent wheezing and asthma [82,84].

Several studies have assessed the long-term respiratory problems of late preterm infants. In a study published in 2013 on a Dutch cohort, there was noticed a double number of hospitalizations in moderate-preterm children compared to term neonates [85]. The differences were maintained also at preschool age (more coughing or wheezing during a cold or without a cold, and more use of medication in the moderate-preterm neonates) [85]. Risk factors for these symptoms in the category of moderate-preterm infants were also identified: passive smoking, a family history of asthma and high social class [85]. A cohort study performed in Canada obtained the same results: late preterm neonates had significantly greater adjusted odds for lower respiratory tract infections in the preschool years and asthma at school age compared to children born at term—this study also identified several associated risk factors [86]. In a study on late preterm patients followed until adolescence, the formerly late preterm children had a slightly higher incidence of coughing and hay fever and a slightly lower peak expiratory flow compared to their term controls [87]. Regarding the adult respiratory outcomes of the former late preterm infants, a review of the medical literature failed to show an increased risk of asthma or allergy in this category compared with children born at term [88]. This paper should, though, be regarded with caution because the studies cited included mostly cohorts of certain populations (Northern European countries) [88]. It was established, though, that late preterm and early term infants have an increased early adult all-cause mortality [88].

Regarding the risk of RSV infection, the rates of hospital admissions for RSV infections is similar between infants born at 33–35 weeks gestational age and infants born at less than 32 weeks [89]. A review of two studies showed that the risk of severe RSV infections in infants born at 33–35 weeks gestational age is similar to that of infants less than 32 weeks gestational age [90]. The odds ratio for bronchiolitis-related death in 33–35 weeks infants compared to infants born at term is 5 [91]. As stated above, RSV infection in late preterm infants without atopic predisposition represents a risk factor for the subsequent development of asthma [92]. Considering all these risks, it was suggested that late preterm infants should receive palivizumab prophylaxis based on a risk factor approach [93].

A Canadian study [93] effectively used a risk scoring tool to select neonates with gestational ages 33–35 weeks for receiving palivizumab prophylaxis. The scoring tool consisted of seven items that were attributed values according to their importance.

Infants with scores higher than 49 (moderate and high risk) were eligible for prophylaxis. As can be seen, the risk factors are represented in the order of importance by birth during the peak of RSV season, siblings in daycare, crowding SGA and male status of the patient, and more than 1 smoker in the house. Using this score, 19% out of this population of premature infants was selected to receive prophylaxis. The score is based on a previous study performed by a research group in Spain [90].

As can be seen, this category of former premature infants, forgotten in almost all follow-up programs, has important risk factors and important pathologies during infancy and childhood, both comparable to the early (less than 32 weeks) former premature infants. All late preterm neonates should be examined at discharge from the point of view of the respiratory risks. The patients that are included in the neurologic follow-up program will also be followed from the respiratory point of view [4]. Because prematurity represents a chronic condition that implies risks for the development in several domains, including the respiratory development and risks [8]. This fact should be mentioned in the discharge notes for late preterm neonates, in order to make the general pediatricians or family physicians aware regarding the respiratory risks for this category of neonates (see above).

## 7. Rationale and Framework for a Respiratory Follow-Up Program for the Former Premature Infants

The previous sections showed that the former premature infants are at risk for respiratory pathology due to factors related to prematurity. They present with a greater risk of respiratory infections, and especially of severe forms of bronchiolitis, than children born at term. They also present with lower than normal respiratory volumes and flows, and developmental lung and airways abnormalities, and this leads to an increased risk of airways disorders (recurrent wheezing and asthma). The sequelae are present from early infancy throughout childhood and until adult life.

Based on these arguments, a respiratory follow-up program for former premature infants is perfectly justified. It fulfils all the roles of a follow-up program (as stated in one of the above sections):

- Support for the child—identifies from the maternity hospital a population at risk that could be followed and allows establishing preventive measures for populations at risk—RSV prophylaxis for selected groups of patients, avoidance of triggers for patients at risk of asthma, etc. Periodic respiratory examination and pulmonary function tests could allow early identification of the children with a risk of wheezing and asthma and early treatments. According to the AAP policy statement in 2014 (still valid) RSV prophylaxis for palivizumab is indicated for two categories of preterm neonates, during the first year of life:
- Premature infants born before 29 weeks 0 days of gestation
- Preterm infants with chronic lung disease defined as birth <32 weeks 0 days, and requirement for >21% oxygen for at least 28 days after birth [94].

As another indication, the 33–35 weeks neonates selected to be at risk according to the tool quoted previously regarding the late preterm infants (see the previous section regarding late preterm infants).

- Support for the families—the program allows a partnership with the families. The respiratory risks could be explained to the family at discharge from the hospital (a flyer could be developed as in the cases for the neurologic follow-up). The family could be helped to avoid behaviours at risk both for the family (smoking in the family, crowding) and for the patient (smoking, avoidance of sports) and encouraged to maintain a good hygiene with avoidance of infections and allergens. Moreover, knowing the respiratory status of the child would be of help in decisions about the practice of certain activities and sports
- Support for the healthcare system: a good follow-up program could offer data as complete as possible regarding the respiratory outcome of former premature infants, allowing the stakeholders to manage the levels of care and services available. The data could also help as a feedback for the units, in order to try to correct risk factors that lead to unfavorable outcomes, if possible

Even if the medical literature states the need for the inclusion of a respiratory follow-up component in the follow-up program for premature neonates [4], there is no consensus yet about the structural framework of the program. All the program suggest a history regarding the respiratory risks and symptoms and a clinical examination of the respiratory system as a part of the evaluation [4]. As an example of a case used in investigating the respiratory component of the outcome of former premature infants, the Prematurity and Respiratory Outcomes Program (PROP) [9], a study protocol published in 2015, offers a comprehensive file for data collection that could be used for this purpose reference [9], additional file 5 Other papers suggest other examination scores for assessing the patients in the program, for example The Respiratory Distress Assessment Instrument, the Respiratory Assessment Change Score (RACS) [13], the International Study of Asthma and Allergies in Childhood (ISAAC) Questionnaire [4,14] or the 6 min walk test [4,15]. The main problem of these tests is that each one is specific for a certain age (infants and toddlers—RACS [13] or children over 6 years—ISAAC Questionnaire, 6 min walk test [14,15]) so the results are not comparable. Things are not the same in the case of the neurologic follow-up, where the neurological examinations used—Amiel Tison [16] ore Prechtl [17]—have the same items assessed at different ages and comparisons regarding development can be made. Regarding pulmonary function tests, as explained in a previous section, they could be performed in older children and in special labs. In conclusion, the safest approach would be, in our opinion, a comprehensive history using a standardized form and a respiratory clinical examination performed at each visit.

The structure of such a respiratory follow-up program could be linked to a regular neurologic and neurodevelopmental program [4,5]. The first examination would occur at 40 weeks corrected age or at discharge from the maternity hospital [4,5]. All the premature infants in the follow-up program have a starting visit at 40 weeks corrected age. At this visit, a respiratory system history and examination should be performed as a start for the respiratory follow up program [4,5]. There will be noted the risk factors for respiratory pathology: gestational age, birthweight, duration of the mechanical ventilation, duration of oxygen need and any antibiotic use (for references see above). Additionally, data should be gathered about familial and environmental risk factors (smoking, atopy and asthma in the family, siblings with acute and chronic conditions, socio-economic status). The family should be informed about the risks and a partnership should be established. The patient will receive a full clinical examination of the respiratory system. The visits should be performed more often during the first 2 years of life (at 4 months interval during the first year and at 6 months during the second year), then yearly until 6 years of age. The history and examinations could be performed by the neonatologists in charge of the follow-up program. In cases where abnormalities are detected, the child should be referred to the pediatric pulmonologist. In the case of infants with more serious chronic conditions (bronchopulmonary dysplasia/chronic lung disease) the follow up should be performed by the pediatric pulmonologist. At this age, a full evaluation of the pulmonary function tests is possible, as well as establishing a risk for asthma. In our view, there should be two examinations in the respiratory follow-up program (at 2 years and 6 years) that should be performed in cooperation with the pediatric pulmonologist, in order to establish the risk of asthma and the need for respiratory function tests). This could be an important visit for counselling the family about the future directions in the development of the child and special precautions to be taken. The visits could be rarer from this moment, but a visit needs to be performed together with the adult pulmonary specialist before 18 years old, in order to discuss together the risk and the continuation of the follow-up until adulthood.

As in the case of the neurologic follow-up, the respiratory follow-up program should not only have a diagnostic component, but also a component of early intervention. There should be established links with pediatric emergency departments, pediatric infectious disease specialists and pulmonary function laboratories and clinics specialized in the monitoring and treatment of asthma. Moreover, information could be provided to the general practitioners in order to improve the care of their patients. Table 4 summarizes the structural framework of a respiratory component of a follow-up program for children born preterm and admitted in the NICU (neonatal intensive care unit).

## 8. Conclusions

The former premature infants present, due to lung immaturity at birth, extrauterine development of the respiratory system, pulmonary conditions in the neonatal period and deficient control of breathing, and a risk of medium- and long-term pulmonary problems. This consist of respiratory tract infections (mainly bronchiolitis caused by viruses such as rhinoviruses and respiratory syncytial virus) during the first year of life, airway diseases such as recurrent wheezing and asthma during childhood and abnormalities of the lung function that could place them at risk for complications later in life. This is why a respiratory follow-up program is needed for every NICU as is the case for the neurodevelopmental follow-up program. Establishing such a program could lead to the early detection of risks and preventive therapies put in place in order to improve their prognosis and ensure a normal and productive life. 

## Figures and Tables

**Table 1:** Risks factors for pulmonary and respiratory sequelae in the premature neonates [8,18,19,20,21,22,23].

Extrauterine development of the lung
Canalicular stage (16–26 weeks gestational age)
Saccular stage (24–38 weeks gestational age)
Alveolar stage (36 weeks gestational age—3 years postnatal age)
Pulmonary pathology in the neonatal period
Specific to the premature neonate
Respiratory distress syndrome
Transient tachypnea of the newborn
Non-specific
Pneumonia
Air leak syndromes
Control of breathing
Apnea—obstructive; central

**Table 2:** Physio-pathologic scenarios in wheezing disorders of premature babies [54,55].

Structural Disease Scenario	Active Disease Scenario
Mechanism: altered elastic and fibrous networks	Mechanism: chronic inflammation
Possible causes: - Hyperoxia - Positive pressure ventilation	Possible causses: - Deficient immune response - Th2 shifting - Stress - Premature chemosensory stimulation

**Table 3:** Methods of evaluating pulmonary function in children [68,69,70,71,72].

Technique	Advantages	Disadvantages
Spirometry	Standardized Reproductible Can measure a large number of respiratory parameters Can establish a diagnosis of type of airway dysfunction	- Can be performed in an older, cooperative child - A rather large number of factors that can modify the results and need to be recorded or controlled
Tidal breathing analysis	Can be performed in an infant Easily available equipment	Non-standardized Lacks proper reference values
Raised volume rapid thoracoabdominal compression	Can be performed in an infant Determines a large number of volumes	Used in a few highly specialized centers around the world
Whole body plethysmography	Can be performed in an infant Can determine functional residual capacity Non-standardized	Only for research purposes

**Table 4:** Proposed structural frame for a respiratory follow up program.

	40 Weeks Corrected Age	4 Months *	8 Months *	12 Months *	18 Months *	24 Months *	3 Years	4 Years	5 Years	6 Years	14 Years	18 Years ****
Clinical examination												
Anthropometric indices												
Clinical exam respiratory system												
Control at pulmonologist **	***											
Pulmonary function test **												

Legend: mo—months; Y—years; Anthropometric indices—head circumference, thoracic circumference, weight, height, body mass index. * Corrected ages (age counted from 40 weeks post-menstrual age of the patient). ** The physician in charge of the respiratory follow up could indicate a control to the pulmonologist in the case of abnormalities of the clinical examination or repeated episodes of wheezing or respiratory tract infections. *** In the case of patients with chronic lung disease/bronchopulmonary dysplasia, the first control at the pulmonologist will be at 40 weeks corrected age. **** At the age of 18 years, the patient will be referred to the adult pulmonologist. The grey areas represent the ages at which different examinations and tests are performed.

## Data Availability

Not applicable.
